# Reconstruction of delayed diagnoses simultaneous bilateral distal biceps tendon ruptures using semtendinosus and quadriceps tendon autografts

**DOI:** 10.1186/s40064-015-0897-7

**Published:** 2015-03-07

**Authors:** Lars Blønd, Bo Kaewkongnok

**Affiliations:** Teres Hospital Parken, Øster Alle 42, 3, DK-2100 Copenhagen, Denmark; University Hospital of Køge, Lykkebækvej 1, DK-4600 Køge, Denmark

## Abstract

**Introduction:**

Simultaneous bilateral distal biceps rupture is rarely reported, but several issues has to be taken into account and the here presented case brings up some of these aspects.

**Case description:**

This case presents a simultaneous bilateral distal biceps ruptures, and due to delayed diagnosis, bilateral muscle retraction had occurred. Surgical reconstruction was challenged both by social circumstances and by previous harvesting of the Semitendinous tendon on one side. The surgery was performed as a staged approach, using a Semitendinosus graft at one side and later using a Quadriceps tendon graft on the opposite site. At follow-up 14 month postoperatively the Oxford Elbow Score was 92 percent for both elbows and the MRI’s bilaterally demostrates that the grafts are tight.

**Discussion and evaluation:**

The Quadriceps tendon is a previously non reported type of graft material for this type of surgery. Based on the experience from this case it is concluded that six weeks after trauma the degree of retraction of the distal biceps tendon can impair direct repair.

**Conclusion:**

When reconstruction of the ruptured distal biceps tendon is needed, subjective normal forces can be obtained using both the semitendosus graft as well as the quadriceps graft, however with a minor increased donor site morbidity with respect to the quadriceps graft.

## A case report

Simultaneous bilateral distal biceps rupture is rare. It is generally accepted that early surgical treatment is recommended for distal biceps rupture, since the outcome of conservative treatment often results in reduced strength and negative consequences for activities of daily living and especially more strenuous activities (Baker and Bierwagen 1985; Dillon and King 2013). In most cases the ruptured distal biceps tendon can be repaired by primary reinsertion, however after some weeks, the tendon itself can be retracted and by this in combination with the formation of fibrous scar tissue, the repair can be precluded. These more chronic cases can be addressed by bridging the gap between the retracted biceps muscle and the radius bone with a reconstruction using either autograft or allograft tissue. Autograft materials such as the semitendinosus tendon (Hallam and Bain 2004; Hang et al. 1996; Wiley et al. 2006), the tensor fascia lata (Bayat et al. 1999; Ryhänen et al. 2006), the flexor carpi radialis tendon (Levy et al. 2000), palmaris longus (Ryhänen et al. 2006; Vastamäki and Vastamäki 2008) and the long extensors of second and third toes (Vastamäki and Vastamäki 2008) and autograft materials such as the achilles tendon (Snir et al. 2013), has been described and good results has been reported.

Four cases with simultaneous distal biceps ruptures have been published (Bayat et al. 1999; Rokito and Lofin 2008; Dacambra et al. 2013; Bell et al. 2000). In two cases late reconstruction using tendon grafts were needed. The here presented case had simultaneous bilateral distal biceps ruptures, and due to delayed diagnosis, bilateral muscle retraction had occurred. We will account that surgical reconstruction was challenged both by social circumstances and by previous harvesting of the semitendinous tendon on one side. The surgery was performed as a staged approach, using a semitendinosus graft at one side and later using a quadriceps tendon graft on the opposite site. The quadriceps tendon is a previously non reported type of graft material for this type of surgery. Based on this special case we want to discus some of the aspects of chronic distal biceps tendon ruptures.

### Case

47 year old fit and healthy male, who is an independent dealer of furniture, and this includes dayly moderate strenuous activities lifting the furniture. For sporting activities he used to do a kayaking, here among competing in a 70 km kayak marathon and bicycling, here among competing in La Mamotte, a 174 km race in the Alps. Except from previous reconstruction of the ACL he had no medical history, and took no medication, non smoker and no history with alcohol abuse. He indicated that he had not previous experienced symptoms from his elbows. In a working situation he had to lift a heavy pallet with left arm and suddenly he felt a snap with subsequent loss of strength and immediately after he switched over to the right arm and felt the same snap in this arm. The patient noted that both biceps muscles were dislocated a little higher than normal. After three weeks he was motivated from his physiotherapist to have a medical examination, and this confirmed the diagnosis of bilateral distal biceps rupture. At inspection the biceps muscles were displaced proximal and a positive hooks test were observed. Moreover a reduced elbow flexion and supination forces grade 4, with normal active range of movement was found. The patient was determined to get both tendons made simultaneously. After having discussed the postoperative immobilization regime using a sling with no permission to do active elbow flexion he realized that this was not practically any option based on his social circumstances living alone. Decisions were made to operate in a staged manner doing the dominant right biceps tendon primarily, and secondary the left biceps after a four weeks delay. The operative planning was challenged by previous harvesting of the hamstrings tendon for the ACL reconstruction in his left knee.

### Operation right arm 6 weeks after injury

Incision distal to the antecubital fossa (single incision technique) and a concomitant incision more proximal to the fossa, the lacertus fibrosus was found ruptured and the biceps tendon was dissected and found retracted approximately 8 cm. After extensive mobilization by blunt finger and scissors dissection around the m. biceps brachii, a tendon gap of 3 cm to the tuber radii, in combination with a thin tendon sustained with a longitudinal rupture as well, prevented primary repair. From the ipsilateral lower limb a good quality semitendinosus tendon graft was harvested. The tuberosity radii foot print was debrided and a central placed 5 mm metal Corkscrew anchor loaded with double Fiberwire (Arthrex Inc., Naples, FL, USA) sutures was inserted and the graft was sutured firmly to footprint and then merged into the biceps tendon and secured in the correct length, by evaluation of the tension in fully elbow extension and pronation, using Orthocord #2 suture.

### Operation left arm 10 weeks after injury

Similar exposure and technique was used as in the right arm and also the preoperatively findings were equal compared to the left arm. With no easy access to allograft tissue and with a lag of hamstring graft based on previous harvesting bilaterally, it was decided to use the ipsilateral quadriceps tendon. A 8 mm wide, 4 mm thick and 8 cm long full thickness quadriceps tendon graft was harvested and reinserted corresponding to the technique described for the right arm.

### Post operatively rehabilitation protocol

Both elbows were subject to the same rehabilitation protocol. The elbow was unloaded by a 90 degrees solve universal arm sling for four weeks. Guided full range of movement with passive exercises and active extension and pronation exercises for the elbow and forearm was allowed from day one postoperatively and physiotherapy was started after one week. Active exercises for elbow flexion against gravity and forearm supination, was allowed after 4 weeks. A maximum of 2 kg load after 6 weeks and a maximum of 10 kg load were allowed after 10 weeks.

## Results

**Figure 1:**
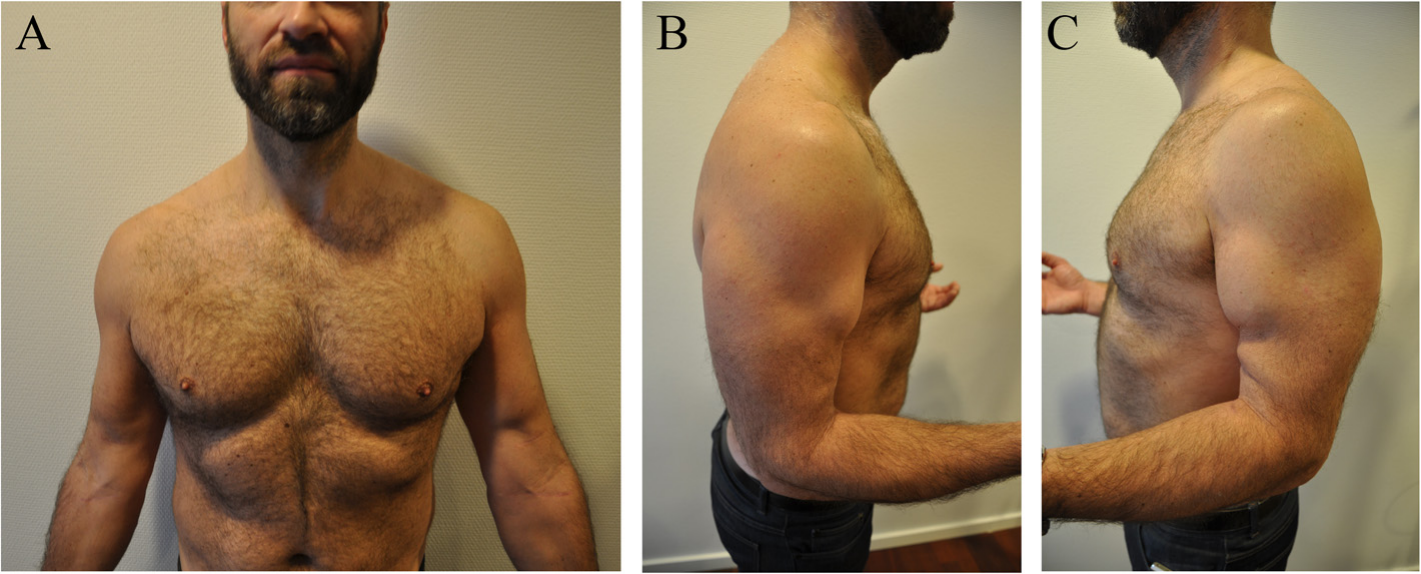
**A,B,C these pictures demonstrates the results 4 and 5 month postoperatively, with bilaterale high placed biceps during active muscle contraction.**

**Figure 2:**
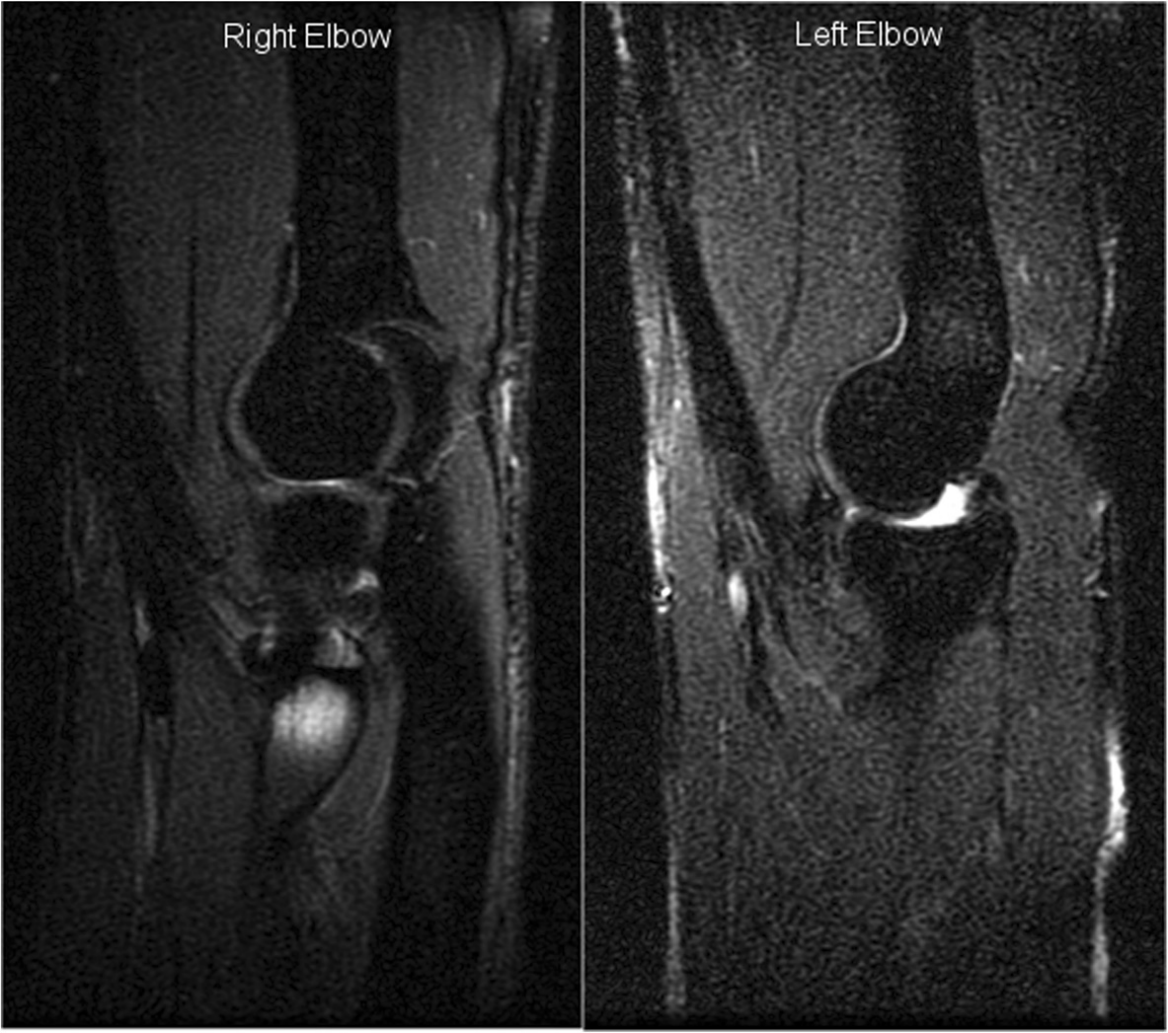
**Postoperative MRI at 14 month follow-up demonstrate well proportioned and tight grafts bilaterally.** Right elbow is semtendinosus graft and left elbow is quadriceps graft.

At 4–5 month follow-up, normal range of movement had been achieved and muscle strength was subjectively recovered. The biceps muscle belly appeared displaced a few centimeters proximal bilaterally. See Figure 1a, b and c. He report that the donor site morbidity has been significantly less from the semitendinosus donor site compared to the quadriceps donor site and especially for the first 6 month postoperatively, he experienced significantly more pain in the quadriceps area compared to the opposite hamstrings area, and this pain affected his physiotherapeutic progression and the level of sports activity when bicyling. At 12 month follow-up he has obtained normal sports activities, but he experienced minor sore from the quadriceps donor site. At follow-up 14 month postoperatively the Oxford Elbow Score was 92 percent for both elbow and the MRI’s bilaterally demostrate that the grafts are tight. See Figure 2.

## Discussion

From this case we have demonstrated that good results can be obtained by delayed reconstruction of distal biceps ruptures, both by using semitendinosus and by using quadriceps grafts. The patient presented achieved full range of movements and regained elbow and forearm strength. Regardless of whether a repair or reconstruction is warranted, you have to decide if to repair either both tendon ruptures at the same time, or to do it in a staged manner. Several factors must be considered such as the patients’ social and occupational situation, hand dominance, medical comorbidities and general health. In cases with simultaneous repair or reconstruction, the mobility of the arms are reduced based on the postoperative regime, that normally restricts active flexion and supination for a 2–6 weeks, according the surgeons preferences. With the staged approach the patient is allowed complete use of the non-operated arm for activities of daily living while convalescing from surgery of the opposite arm. There is currently no evidence to guide this, and the decision should be made in collaboration with the patient. From the literature it seem that comparable good results can be obtained for both acute tendon reinsertion and for later reconstruction of the tendon gap (Wiley et al. 2006; Bayat et al. 1999; Snir et al. 2013; Rokito and Lofin 2008; Dacambra et al. 2013; Nesterenko et al. 2010).

Complete ruptures of the distal biceps tendon are classified as acute or chronic, depending on the time period between injury and diagnosis. Ruptures occurring after 4 weeks are generally considered chronic (Bell et al. 2000; Ramsey 1999). However, authors have used a range of time intervals, from 3 to 12 weeks, to define chronic tears (Darlis 2006; Kaplan 2002; Kelly et al. 2000; Sanchez-Sotelo et al. 2002). Chronic ruptures are further subdivided based on the integrity of the bicipital aponeurosis (lacertus fibrosus). An intact lacertus fibrosus prevents tendon retraction proximally, thereby increasing the possibility of a late primary repair (Ramsey 1999). In our case we found, ruptures of both the aponeurosis and the tendons retracted to a degree that impaired primary reconstruction six weeks after injury, thus confirming the observations from others. The biceps brachii is the primary supinator and a major flexor of the arm at the elbow. Ruptures of the distal biceps have been shown to results in a loss of 8% to 36% of flexion strength and 21% to 55% of supination strength, which may be debilitating for sports performance as well as activities of daily living (Baker and Bierwagen 1985; Mariani et al. 1988; Nesterenko et al. 2010). Residual weakness, loss of endurance strength, chronic pain, and disability are well documented in the natural history of untreated ruptures of the distal biceps (Ramsey 1999; Baker & Bierwagen 1985; Nesterenko et al. 2010). There are four cases in the literature which describe simultaneously distal biceps rupture with almost equally good results. DaCampra et al. (2013) describes a 43 year old male lifting a large sheet of drywall and first he felt a “pop” and pain in his right elbow, then shifting to the left arm and had the same experience and distal biceps tendon ruptures bilaterally was diagnosed. A staged procedure was chosen with a 6 weeks interval, and in both cases primary repair were achieved. The patient returned to his normal duties with mild congestive antebrachial cutaneous nerve injury. Subjective he had 85% of his normal functioning. Rokito et Lofin (2008) reported on a male 51 -year-old recreational weightlifter who ruptured both distal biceps tendon when he did preacher curls. His dominate right arm was first operated without tendon graft six weeks post injury. The other arm was reconstructed using achilles tendon allograft 13 weeks later. Subjective he regained full strength and was able to weight lifting 6 months after. Bayat et al. (1999) reported on a 50-year-old rock climber sustaining bilateral injuries during mountain climbing, initially treated conservative. Due to lack of flexion and supination force, he had staged tendon reconstructions with ipsilateral fascia lata, 2 years after injury, with a six month interval. Subjective he obtained a satisfactory results. Bell et al. (2000) describes the outcome of distal biceps rupture in 26 male patients with three different types of treatments. Respectively conservative, reinsertion and reinsertion with allograft. One patient had a simultaneous bilateral rupture, but there is not a clear description of the type of treatment the patients completed and what the outcome was. Wiley et al. (2006) compared 7 patients with late reconstruction to 7 patients treated conservatively and found that flexion and supination strength was restored in the operated group, while a 20% reduced strength was found in the conservatively treated group. Late reconstruction for chronic ruptures of the distal biceps using allograft tissue can as well be an safe and effective solution (Snir et al. 2013; Mascarenhas et al. 2009). In our here presented case the first choice of graft was the semitendinosus tendon graft. This was based on previous good experiences, the recommendations from the literature (Hallam and Bain 2004; Hang et al. 1996; Wiley et al. 2006) and the fact that we are used to harvest this graft doing ACL surgery. Given that the semitendinousus tendon graft on the other site has been harvested after previous ACL reconstruction we had to look for another graft material. We made the same practical approach based on our experience from ACL surgery and therefore selected the quadriceps graft, a graft with good biomechanical properties together with low donor site morbidity (Lund et al. 2014). Typically a graft length of 8 cm and 9–10 mm wide, can be obtained and this can match the diameter of the distal biceps tendon of approximately 9 mm for men (Cho et al. 2011).

## Conclusion

Based on the experience from this case we can conclude that six weeks after trauma the degree of retraction of the distal biceps tendon can impair direct repair. Subjective normal forces can be obtained using both the semitendinosus graft as well as the quadriceps graft, however the quadriceps graft gives a minor increased donor site morbidity the first year.

## Consent

Written informed consent was obtained from the patient for the publication of this report and any accompanying images.
